# Leakage-Free Multimodal Depression Screening: Controlled Evaluation of Text, Facial Behavior, and Prosodic Fusion

**DOI:** 10.3390/bioengineering13091009

**Published:** 2026-08-30

**Authors:** Souaad Hamza-Cherif, Nesma Settouti

**Affiliations:** 1 Biomedical Engineering Laboratory, University of Tlemcen, Tlemcen 13000, Algeria; 2 LabISEN, LSL, ISEN Ouest, 14000 Caen, France

**Keywords:** depression screening, DAIC-WOZ, E-DAIC, multimodal learning, behavioral sensing, data leakage, participant-level evaluation, late fusion, prosodic features, explainable AI

## Abstract

Multimodal behavioral sensing may support depression screening, but evaluation on small clinical-interview datasets is particularly vulnerable to data leakage and model-selection bias. We present a leakage-audited trimodal framework evaluated on DAIC-WOZ (n=180, PHQ-8 ≥10), combining SBERT text embeddings, OpenFace facial-behavior descriptors, and COVAREP prosodic features. Participant-level partitioning is performed before augmentation, while decision thresholds and neural-model checkpoints are selected exclusively from internal validation data. A controlled five-seed experiment showed that a deliberately leaky full-pool MixUp construction, in which a retained development sample could include a held-out participant as its second parent, was associated with a 27–33 percentage-point increase in Macro-F1 across four fusion configurations. Under the participant-level leakage-free 5-fold protocol, trimodal late fusion achieved a Macro-F1 of 0.532±0.041, compared with 0.446±0.021 for Text+Imaging late fusion. Paired participant-level correctness outcomes also favored trimodal fusion (McNemar $\chi^{2}=6.618$, p=0.010), consistent with improved paired classification when the audio modality was included under the leakage-free protocol. On 86 participant-disjoint E-DAIC sessions, using a consistent PHQ-8-based outcome definition (PHQ-8 ≥10), trimodal late fusion achieved Macro-F1 = 0.621 and AUC = 0.676; this experiment is interpreted as within-family generalization rather than independent cross-corpus validation. Overall, the results show that leakage control can substantially alter both absolute performance and comparative conclusions, and that multimodal gains should be established through participant-level evaluation, modality-specific analysis, and reproducible model-selection procedures.

## 1. Introduction

Major depressive disorder (MDD) affects over 332 million people worldwide and ranks among the leading causes of disability [1]. Despite its prevalence, depression remains frequently underdiagnosed, partly because access to mental-health specialists is limited in many regions, particularly in low- and middle-income countries. Validated self-report instruments such as the PHQ-8 [2] provide practical first-line screening tools, but their use still requires appropriate clinical integration and does not fully address the need for scalable screening support in digital or telemedicine contexts. Automated computational screening tools may therefore serve as a complementary aid to clinical assessment, provided that they are developed, validated, and interpreted under methodologically sound conditions.

The past decade has seen growing interest in multimodal approaches that combine linguistic, visual, and prosodic signals extracted from structured clinical interviews. Depression can affect multiple behavioral channels simultaneously: speech may become flatter, slower, or more monotonous; facial expressivity may be reduced; and language may reflect negative affect, social withdrawal, or psychomotor slowing. This multi-channel nature motivates the integration of complementary modalities into a single framework while also raising methodological questions about how such modalities should be combined and evaluated.

From a bioengineering perspective, the framework is positioned as a multimodal behavioral-signal fusion and computer-aided screening system. It integrates prosodic and voice-related descriptors derived from speech using COVAREP (Section 3.2.3), session-level facial landmark and gaze descriptors extracted with OpenFace, and linguistic representations derived from participant interview transcripts (Section 3.2.1). The engineering contribution lies in evaluating how these heterogeneous behavioral signals can be processed and fused reproducibly for computer-aided depression screening; the resulting system is intended as decision support rather than as a substitute for clinical diagnosis.

Among publicly available benchmarks, the DAIC-WOZ dataset [3]—a corpus of semi-structured clinical interviews between participants and the virtual agent Ellie—has become a widely used testbed for computational depression detection. However, reported performance figures on this benchmark must be interpreted cautiously. A recent systematic review of 536 DAIC-WOZ studies found that only a small fraction met minimal reproducibility standards, with data leakage, including augmentation before participant-level splitting, identified as a major methodological flaw [4]. Such leakage can allow information from test participants to influence the training process and may substantially inflate reported performance on nominally unseen data.

This paper addresses this issue by designing and evaluating a trimodal depression-screening pipeline under a strict leakage-free protocol. The proposed framework integrates SBERT text embeddings, OpenFace 2.0 behavioral imaging features, and COVAREP prosodic features. Participant-level splitting is enforced before any augmentation, and the primary evaluation is performed using leakage-free 5-fold cross-validation on the full 180-participant DAIC-WOZ cohort. In addition to reporting internal performance, we quantify the impact of a leaky augmentation protocol, analyze modality contributions using model-agnostic grouped permutation with SHAP/LIME as secondary diagnostics, evaluate screening-relevant clinical metrics such as sensitivity, and assess partial generalization on a disjoint extended E-DAIC cohort. The evaluation protocol additionally prevents model-selection leakage by restricting decision-threshold optimization and neural early-stopping/checkpoint selection to internal validation data. Section 3.3, Section 3.4, Section 3.5 and Section 4 describe the protocol and the controlled augmentation-leakage results.

The contributions of this paper are as follows.

First, we quantify the performance inflation associated with augmentation-partition leakage through a paired construction-level comparison of leakage-free sub-training-only MixUp and a deliberately leaky full-pool construction. In the leaky condition, a stratified train/test split is defined first, but MixUp parents are then sampled from all participants, and a synthetic observation is retained for development solely according to the partition membership of its first parent; its second parent may therefore be a held-out test participant. Across four bimodal/trimodal early- and late-fusion configurations and five independent seeds, this partition-violating construction is associated with a 27–33 percentage-point increase in Macro-F1 relative to the leakage-free comparator.

Second, we evaluate a trimodal Text+Imaging+Audio framework under a participant-level 5-fold protocol in which preprocessing, augmentation, checkpoint selection, and threshold selection are restricted to development data. Trimodal late fusion achieves Macro-F1 =0.532±0.041, compared with 0.446±0.021 for Text+Imaging late fusion, a mean fold-level difference of +0.086. On pooled held-out predictions from all 180 participants, paired correctness outcomes favor trimodal fusion under McNemar’s test ($\chi^{2}=6.618$, p=0.010); this test evaluates classification discordance rather than the Macro-F1 difference itself.

Third, we examine whether the gain is consistent with modality-specific evidence. Text+Audio late fusion reaches Macro-F1 =0.481±0.055, exceeding Text+Imaging late fusion by +0.035, while the two strongest overall configurations are trimodal LightGBM early fusion (0.542±0.025) and trimodal late fusion (0.532±0.041).

Fourth, we add a model-agnostic grouped-modality permutation analysis on the two strongest trimodal classifiers: LightGBM early fusion and late fusion. Using 500 permutations per modality while keeping the validation-selected decision threshold fixed, the analysis quantifies the mean decrease in Macro-F1 and AUC when each modality is disrupted. For LightGBM, imaging produces the largest AUC decrease (ΔAUC=0.057), whereas text produces the largest Macro-F1 decrease (ΔF1=0.039). SHAP and LIME on XGBoost are retained only as secondary, model-specific diagnostics.

Fifth, we report participant-level screening metrics pooled across the five outer test folds. Trimodal late fusion obtains Macro-F1 =0.537, sensitivity =0.411, specificity =0.669, PPV =0.359, and NPV =0.716 on the pooled predictions.

Sixth, we provide a participant-disjoint evaluation on 86 E-DAIC sessions represented in a homogeneous E-DAIC-native feature space. For all E-DAIC analyses, depression status is defined consistently from the raw PHQ-8 score using the study threshold PHQ-8 ≥10. This PHQ-8-based definition applies to 20 of the 275 pooled E-DAIC records for which the official binary label indicates non-depression despite a raw PHQ-8 score ≥10. Under this consistent outcome definition, trimodal late fusion obtains Macro-F1 =0.621 and AUC =0.676. Because DAIC-WOZ and E-DAIC share the same corpus family and related acquisition context, this experiment is explicitly treated as within-family generalization rather than independent cross-corpus validation.

The remainder of this paper is organized as follows. Section 2 reviews related work on unimodal and multimodal depression detection, data leakage, and explainability. Section 3 describes the dataset, feature extraction pipeline, evaluation protocol, model architectures, and explainability approach. Section 4 reports the experimental results, including the participant-disjoint E-DAIC generalization analysis. Section 5 discusses the findings, limitations, and future directions. Section 6 concludes the paper.

## 2. Related Work

### 2.1. Unimodal Approaches to Depression Detection

Automated depression detection has been investigated from linguistic, visual, and acoustic information, with each modality capturing different behavioral correlates of depressive symptoms. Early text-based approaches relied on lexical, affective, and stylistic markers extracted from social media or interview transcripts [5,6]. More recently, contextual language representations based on BERT and related Transformer architectures have increasingly replaced hand-crafted linguistic features [7,8,9]. Domain-adapted models such as MentalBERT [10] further illustrate the potential of representations pretrained on mental-health-related text, while large language models have also been investigated for transcript-based depression assessment [11,12].

Visual approaches exploit behavioral manifestations that may accompany depression, including reduced facial expressivity, altered gaze behavior, and psychomotor changes. Automated facial-analysis frameworks have therefore been applied to facial landmarks, action units, head pose, and gaze signals [13]. OpenFace 2.0 [14] provides a standardized extraction pipeline for these descriptors and has been widely adopted in behavioral-computing studies. Flores et al. [15], for example, investigated temporal facial features for DAIC-WOZ depression screening. However, reported visual-model performance should be interpreted together with the corresponding partitioning and evaluation protocol, which is particularly important when multiple correlated observations originate from the same participant.

Acoustic approaches focus on prosodic and voice-quality characteristics associated with depression, such as changes in fundamental-frequency variability, vocal energy, speaking rate, and voice quality. COVAREP [16] provides standardized low-level descriptors for this purpose. Williamson et al. [17] investigated vocal and facial biomarkers based on motor coordination and timing for automated depression assessment. More recent architectures combine such descriptors with neural temporal modeling. The DAAM framework, for example, reports Macro-F1 values of 0.702 for its CNN–LSTM configuration and 0.720 for its Transformer configuration on DAIC-WOZ [18]. Other work has investigated the relationship between depression-related speech characteristics and speaker-identity representations, highlighting both the potential and the limitations of richer acoustic embeddings [19].

### 2.2. Multimodal Fusion for Depression Screening

Because linguistic content, facial behavior, and speech prosody may encode partially complementary information, multimodal fusion is a natural extension of unimodal depression-screening systems. The general taxonomy of multimodal machine learning distinguishes early, late, and hybrid fusion strategies [20]. Early fusion combines feature representations before classification and can model cross-modal interactions, but it is sensitive to differences in dimensionality, scaling, and representation quality. Late fusion instead combines modality-specific predictions and can be more robust when feature spaces have different statistical properties or discriminative power.

A range of fusion architectures has been evaluated for depression assessment. Al Hanai et al. [21] combined acoustic and textual information using multimodal recurrent networks. Zhang et al. [22] proposed an audio–video–text architecture based on attention mechanisms, reporting a positive-class F1 of 0.76 for trimodal fusion on interview data and 0.78 when predictions from reading and interview tasks were subsequently combined by decision fusion. Nykoniuk et al. [23] compared audio–text early and late multimodal fusion using a modified cohort combining DAIC-WOZ with additional depressive E-DAIC cases, and they reported superior performance for their early-fusion architecture. Their “late-fusion” design retains modality-specific processing branches but subsequently concatenates the resulting representations before joint classification, rather than combining modality-level prediction probabilities at the decision level.

These studies demonstrate the potential of multimodal analysis, but absolute performance values are difficult to compare because datasets, target definitions, feature representations, partitioning strategies, class distributions, and metric definitions differ substantially. Of particular importance, “F1” is not used consistently across the literature: depending on the study, it may denote positive-class F1, macro-averaged F1, weighted F1, or an incompletely specified variant. We therefore preserve the metric definition reported by each source and do not treat F1 values with different definitions as directly comparable.

Methodological audits reinforce the importance of this distinction. Ishikawa and Duke [24] evaluated clinical-interview depression benchmarks under strict subject-disjoint protocols and reported substantial instability in model rankings across evaluation settings, together with weaker transfer to unrelated corpora. Their E-DAIC analysis provides a Macro-F1 of 0.723 under subject-disjoint leave-one-subject-out evaluation. The present study complements this line of work by concentrating on how specific modeling and validation choices within a multimodal DAIC-WOZ pipeline affect the resulting performance estimates, and by examining participant-disjoint transfer within the DAIC/E-DAIC corpus family.

### 2.3. Evaluation Leakage in Clinical-Interview Depression Detection

Data leakage occurs when information that should remain unavailable during model development influences preprocessing, model fitting, hyperparameter selection, or evaluation. In participant-based clinical-interview datasets, this risk extends beyond conventional duplication of training and test observations because many derived samples may originate from the same participant.

One relevant mechanism is augmentation leakage. If augmentation is performed before participant-level partitioning, synthetic observations derived from a participant can contribute to both development and evaluation partitions. This is particularly problematic for procedures such as MixUp, SMOTE, or perturbation-based augmentation because synthetic observations retain information from their source samples. Strict participant-level partitioning must therefore precede any augmentation operation.

A second mechanism is model-selection leakage. Even when participants are correctly separated, test labels must not be used to optimize a decision threshold, select a neural-network checkpoint, determine an early-stopping epoch, tune hyperparameters, or choose among candidate representations. Such operations do not require samples to cross the train–test boundary physically; nevertheless, they adapt the modeling procedure to the nominally held-out outcomes and thereby bias the resulting performance estimate. Model selection must instead be restricted to training data or to an internal validation partition.

Danylenko and Unold [4] systematically examined methodological and reproducibility problems in DAIC-WOZ research and emphasized the importance of clearly documented data partitioning and training procedures. The present study does not claim that data leakage in DAIC-WOZ is newly identified. Rather, it provides a controlled examination of specific leakage mechanisms within a multimodal pipeline and evaluates how conclusions about multimodal performance change once these mechanisms are removed.

Two additional concerns are relevant but conceptually distinct. First, models may exploit systematic properties of interviewer prompts instead of participant-specific behavior, as discussed by Burdisso and Villatoro-Tello [25]. We mitigate this concern by excluding interviewer turns from the linguistic representation. Second, DAIC-WOZ and E-DAIC contain overlapping Wizard-of-Oz participant sessions. Treating overlapping participants as an independent external cohort would therefore constitute participant contamination. Our extended-cohort analysis excludes these shared IDs and retains only participant-disjoint E-DAIC AI-agent sessions.

Together, these considerations motivate an evaluation design in which participant-level splitting precedes augmentation, all preprocessing and model selection are confined to development data, interviewer content is excluded from the text representation, and generalization analyses explicitly account for participant overlap.

### 2.4. Explainability in Multimodal Depression Detection

Explainability can support the analysis of multimodal depression-screening systems by identifying which feature streams contribute to model predictions and whether a classifier is dominated by a single modality. Post-hoc methods such as SHAP and LIME are widely used for this purpose in clinical machine learning research [26]. SHAP provides additive feature-attribution estimates, whereas LIME approximates individual predictions using locally fitted surrogate models. Explainability analyses have also been applied specifically to multimodal mental-health models to investigate acoustic and facial contributions [27].

In the present study, SHAP and LIME are used as complementary diagnostic analyses of early-fusion models. SHAP is applied to XGBoost models to quantify global attribution across text, facial-behavior, and audio feature groups, whereas LIME is used to examine local sensitivity around individual predictions. Because these analyses do not directly explain the final neural late-fusion ensemble, their interpretation is deliberately restricted to modality-level diagnostics rather than to explanations of the final ensemble decisions.

Table 1 summarizes representative studies and, where available, the evaluation protocol and exact definition of the reported performance metric.

The table is intended to provide methodological context rather than a leaderboard. Differences in cohort composition, feature extraction, metric definition, and evaluation design prevent direct ranking of the reported numerical values. The central comparison in this study is therefore performed within the same data and modeling pipeline, with the evaluation procedure varied under controlled conditions.

## 3. Proposed Trimodal Framework

### 3.1. Dataset

We use the DAIC-WOZ dataset [3], which contains semi-structured clinical interviews between participants and Ellie, a virtual interviewer controlled by a hidden human Wizard-of-Oz operator. Each session includes audio recordings, video footage, and full transcripts. Depression severity is measured using the self-reported PHQ-8 questionnaire [2], and we define binary depression as PHQ-8 ≥10, following the threshold commonly used in the literature.

**Figure 1:**
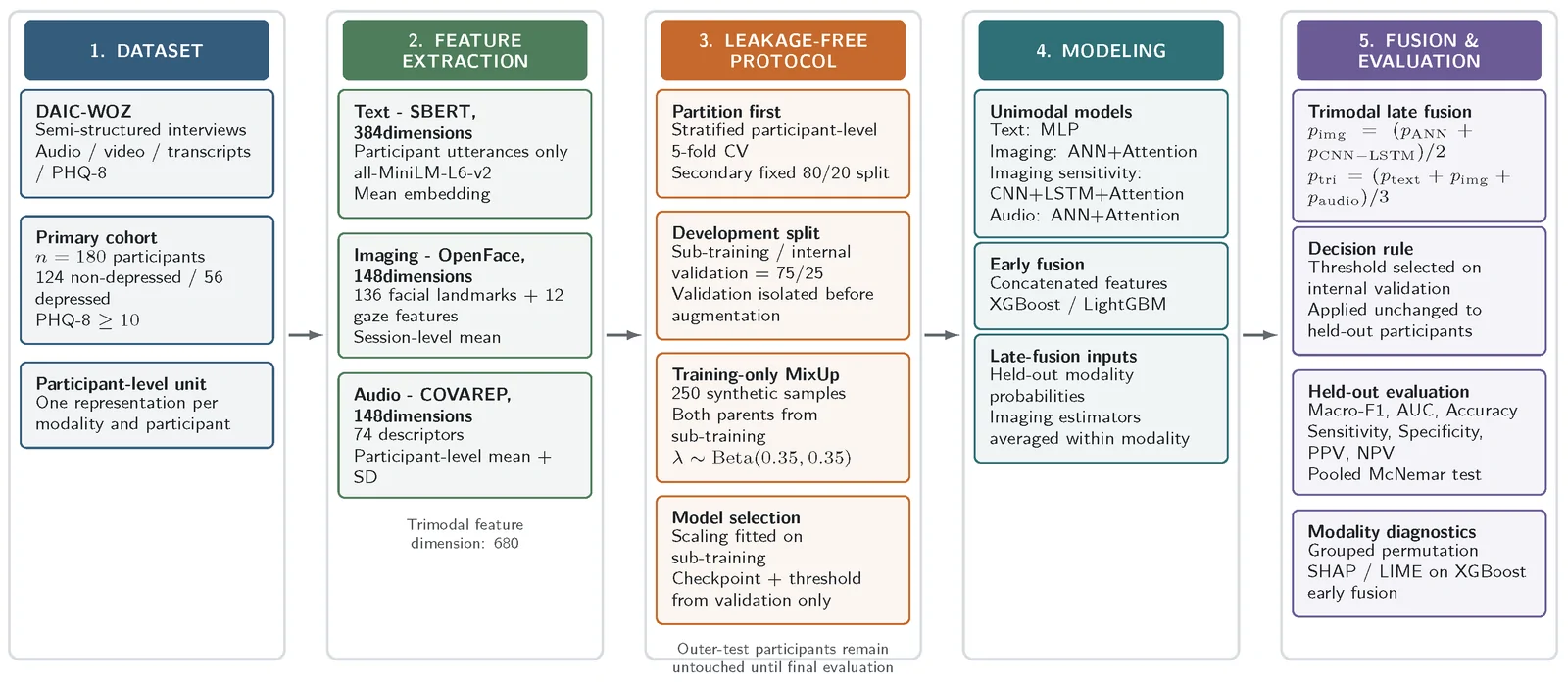
Proposed trimodal pipeline for leakage-free depression screening on DAIC-WOZ. Three participant-level feature streams are extracted independently: SBERT text embeddings (384 dimensions), OpenFace facial-landmark/gaze descriptors (148 dimensions), and COVAREP prosodic descriptors (148 dimensions). Participant-level partitioning is performed before augmentation. Within each outer-fold development set, an internal validation subset is isolated before MixUp; synthetic samples are generated from sub-training participants only. Feature scaling, neural checkpoint selection, and decision-threshold optimization are restricted to development data, leaving held-out participants untouched until final evaluation. The framework compares unimodal models, XGBoost/LightGBM early fusion, and modality-balanced trimodal late fusion, with grouped permutation and SHAP/LIME used as complementary modality diagnostics.

The overall leakage-free trimodal workflow is summarized in Figure 1.

After quality control, removing sessions with corrupted OpenFace extractions, missing audio, or incomplete transcripts, we retain 180 participants: 124 non-depressed participants (PHQ-8 <10) and 56 depressed participants (PHQ-8 ≥10), corresponding to a 31% positive rate. Participant IDs range from 300 to 492.

To ensure traceability across the primary DAIC-WOZ cohort and the E-DAIC-native cohort used for the generalization analysis, Supplementary Table S1 reconciles the cohort definitions, participant counts, class distributions, feature spaces, and documented exclusions used in each analysis. Cohort composition is therefore audited independently of downstream model fitting.

The DAIC-WOZ corpus was collected under ethical approval from its original providers [3]; this study uses the dataset under the access conditions established by USC ICT.

For the generalization analysis (Section 3.9), we additionally use the Extended DAIC (E-DAIC) corpus [29]. E-DAIC reuses Wizard-of-Oz sessions with IDs 300–492 that overlap with the original DAIC-WOZ participant pool and additionally contains participants with IDs 600–718 recorded with a fully autonomous AI-controlled interviewing agent. To avoid participant overlap, shared IDs are excluded from the evaluation cohort, and only the 86 eligible participants with IDs 600–718 are retained.

Because DAIC-WOZ and E-DAIC belong to the same corpus family and share the same general depression-screening setting, this experiment is interpreted as a participant-disjoint within-family generalization analysis rather than as independent cross-corpus validation.

### 3.2. Feature Extraction

#### 3.2.1. Textual Modality (384 Dimensions)

Participant utterances are extracted from the official DAIC-WOZ transcripts. Interviewer turns, corresponding to Ellie’s prompts, are excluded to reduce the risk that the model exploits interviewer-specific patterns rather than participant-level signals, a concern raised by Burdisso and Villatoro-Tello [25]. After text cleaning, including the removal of disfluencies, the normalization of whitespace, and the removal of non-speech markers, sentence-level embeddings are generated using SBERT (all-MiniLM-L6-v2) [30]. Embeddings across all utterances of a given participant are averaged to produce a single 384-dimensional participant-level representation.

SBERT was selected through a preliminary embedding comparison reported in Table 2. All five candidate embeddings were evaluated using the same 3-fold partitions and the same held-out participant set, ensuring that differences in the table do not arise from different evaluation samples. Although several 768-dimensional models provide competitive cross-validation performance, SBERT achieves the highest held-out Macro-F1 while retaining a substantially more compact representation.

This embedding comparison is treated as a preliminary representation analysis rather than as a nested model-selection experiment. Accordingly, the results are used to document the choice of SBERT for the multimodal pipeline but are not interpreted as an unbiased statistical demonstration of its superiority over all alternative encoders. The primary 5-fold results should therefore be interpreted as performance conditional on the fixed SBERT representation, not as a nested comparison of text encoders.

#### 3.2.2. Behavioral Imaging Modality (148 Dimensions)

Frame-level features are extracted using OpenFace 2.0  [14] on all video frames with confidence above 0.5 and a successful detection flag. We retain 68 2D facial landmark coordinates ($x_{0}$–$x_{67}$ and $y_{0}$–$y_{67}$; 136 features) and 3D world-coordinate and head-normalized gaze vectors for both eyes (12 features), for a total of 148 features per frame.

Frame-level values are aggregated by mean over the entire interview session, producing a 148-dimensional participant-level descriptor. Mean aggregation discards temporal dynamics, which is an acknowledged limitation. However, for the session-level binary classification task considered here, it provides a compact representation of average facial and gaze behavior.

The resulting 148-dimensional vector is therefore treated as a tabular participant-level descriptor rather than as a genuine temporal sequence. Architectures operating on this representation are interpreted accordingly; modeling of frame-level facial dynamics is left for future work.

#### 3.2.3. Prosodic Audio Modality (148 Dimensions)

COVAREP [16] acoustic features are extracted from the official USC DAIC-WOZ archive. Each participant’s interview is associated with a COVAREP CSV file (XXX_COVAREP.csv) containing 74 low-level prosodic and voice-quality descriptors sampled at 100 Hz. These descriptors include fundamental frequency (F0), voiced/unvoiced segment probabilities, normalized amplitude quotient (NAQ), glottal-flow spectral tilt (H1–H2), Maxima Dispersion Quotient (MDQ), and spectral-shape parameters (MCEP bands), which have been associated with vocal changes relevant to depression and psychomotor slowing [17].

The extraction pipeline locates the corresponding COVAREP file, filters out silence frames, defined as rows where all 74 values are zero, and computes the per-participant mean (μ) and standard deviation (σ) over all valid frames. This yields a 148-dimensional descriptor:

$$a=\left[\mu ;\sigma \right]\in R^{148}.$$

The mean summarizes the average prosodic state throughout the interview, while the standard deviation captures intra-session variability, including vocal monotony or prosodic richness. Valid frame counts across participants ranged from approximately 8000 to 170,000 (mean ≈85,000), reflecting variable session durations of approximately 10–25 min.

### 3.3. Strict Leakage-Free Evaluation Protocol

The central methodological principle of the evaluation is the strict isolation of held-out participants before any operation that can depend on participant features, labels, synthetic observations, model checkpoints, or decision thresholds.

We implement two evaluation protocols.

#### 3.3.1. Fixed 80/20 Split

The fixed participant-level stratified split uses 144 participants for development and 36 participants for held-out testing. This protocol supports comparison with prior fixed-split results on DAIC-WOZ and provides a stable held-out set for secondary XAI analyses. Because of the small test-set size, fixed-split results are interpreted as secondary analyses.

Within the 144-participant development set, an internal validation subset is created before augmentation and model fitting. The held-out 36-participant test set is not used for model selection, checkpoint selection, decision-threshold optimization, or preprocessing estimation.

#### 3.3.2. Five-Fold Cross-Validation

The primary evaluation uses stratified participant-level 5-fold cross-validation on all 180 participants. At every outer fold, participant partitioning is performed before augmentation, ensuring that no observation derived from a held-out participant can enter model development.

Within each outer fold, the development partition is further divided, stratified by class, into a sub-training set and an internal validation set (75/25). The internal validation set is used exclusively for model-selection operations. Neural-network early stopping and checkpoint selection are based on validation performance, and the decision threshold is optimized on validation predictions over t∈[0.30,0.75] with a step of 0.01. Once the checkpoint and threshold have been fixed, the outer test fold is evaluated exactly once.

Accordingly, no held-out test label is used for feature preprocessing, augmentation, model fitting, early-stopping decisions, checkpoint selection, threshold optimization, or hyperparameter selection. This separation prevents model-selection information from propagating from the evaluation fold into the development procedure.

For reproducibility, participant IDs are sorted deterministically after every multimodal feature merge, and the random-state configuration is fixed before each split and fit. In particular, the PyTorch seed is reset immediately before every neural-model fit so that model initialization does not depend on the order in which preceding models were trained. The complete primary pipeline was executed from two independent full-kernel restarts and produced bit-for-bit identical predictions, selected checkpoints, thresholds, and aggregate metrics.

### 3.4. Training-Only MixUp Augmentation

A MixUp-inspired feature-space augmentation [31] is applied exclusively to the sub-training partition of each fold. The implementation used in the reported experiments differs from canonical soft-label MixUp: features are interpolated continuously, but the interpolated binary target is rounded to a hard class label before training. We therefore describe it explicitly as feature-space MixUp with hard-label assignment rather than as standard soft-label MixUp.

For each synthetic observation, two distinct participants are selected from the sub-training partition only, with one source participant sampled from each class. The same pair of source participants and the same interpolation coefficient λ∼Beta(0.35,0.35) are used for text, imaging, and audio. Given participants *i* and *j*, the synthetic representation for modality m∈{T,I,A} is

$${\tilde{x}}_{m}=\lambda x_{m,i}+\left(1-\lambda \right)x_{m,j}.$$

The label used for model fitting is

$${\tilde{y}}_{\mathrm{hard}}=\mathrm{round}\;\left(\lambda y_{i}+\left(1-\lambda \right)y_{j}\right)\in \left\{0,1\right\}.$$

Neural models are optimized with the standard weighted cross-entropy objective on these hard labels; no soft-label BCE or soft-target cross-entropy is used. Synthetic observations are not assigned an independent participant identity and do not participate in fold assignment. Participant partitioning is completed before augmentation, and every synthetic observation remains exclusively within the sub-training partition from which its two source participants were drawn.

A total of 250 synthetic trimodal observations are generated for each sub-training partition. Feature standardization (zero mean and unit variance) is fitted only on the augmented sub-training data and is then applied unchanged to the internal-validation and held-out outer-test observations. Neither validation nor test observations contribute to estimation of the scaling parameters.

### 3.5. Controlled Augmentation-Leakage Experiment

To empirically quantify performance inflation caused specifically by the interaction between augmentation and participant partitioning, we compare a leakage-free condition with a deliberately leaky condition.

In the leakage-free condition, participant-level partitioning precedes MixUp, and both source participants are drawn exclusively from the corresponding sub-training partition. Consequently, neither parent of a synthetic observation can originate from the internal-validation or held-out test participants.

The deliberately leaky condition is defined as follows. For each seed, a stratified train/test split is first defined with an 80/20 ratio (test_size=0.20, stratify=y, random_state=seed). MixUp generation then ignores this partition boundary and samples from the complete participant index set, i.e., the combined train+test pool. For each of the 250 synthetic observations, two distinct classes are selected; the first parent $i_{1}$ is sampled from one class and the second parent $i_{2}$ from the other. A common coefficient λ∼Beta(0.35,0.35) is used to interpolate text, imaging, and audio features, ${\tilde{x}}_{m}=\lambda x_{m,i_{1}}+\left(1-\lambda \right)x_{m,i_{2}}$, and the synthetic target is assigned as ${\tilde{y}}_{\mathrm{hard}}=\mathrm{round}\left(\lambda y_{i_{1}}+\left(1-\lambda \right)y_{i_{2}}\right)$. The synthetic observation is assigned solely according to the partition membership of the first parent $i_{1}$: if $i_{1}$ belongs to the training partition, the sample is added to the development pool; if $i_{1}$ belongs to the test partition, it is excluded from development. The second parent $i_{2}$ is never used for assignment and may therefore be a held-out test participant. Consequently, a synthetic sample can enter model development while containing a convex contribution from a future test participant. After these synthetic samples have been assigned, the internal validation subset is drawn from this already constructed development pool using a stratified 75/25 split (test_size=0.25, stratify=y_tr_full, random_state=seed). Synthetic observations are not assigned an independent participant identity.

The comparison therefore manipulates the augmentation construction relative to participant partitions rather than the train/test split itself. Within each seed, both conditions start from the same stratified 80/20 participant split and use the same architectures, hyperparameters, candidate augmentation budget ($n_{\mathrm{augment}}=250$), random-seed policy, checkpoint-selection criterion, and decision-threshold rule. The realized number and composition of synthetic observations entering development are not forced to be identical: in the leakage-free condition, 250 synthetic samples are generated within the isolated sub-training partition, whereas in the deliberately leaky condition, 250 candidates are generated from the full participant pool and only candidates whose first parent belongs to the training partition are retained. Consequently, the retained synthetic count in the leaky development pool is seed-dependent and can be lower than 250. Moreover, its internal-validation subset is drawn only after this augmented development pool has been constructed, so synthetic observations can also enter internal validation. The five seeds repeat this paired construction-level comparison independently. We therefore interpret the experiment as quantifying the net performance inflation associated with the partition-violating augmentation construction, not as a perfectly size-matched single-factor ablation. Decision-threshold and checkpoint selection are handled independently using the development-only internal-validation procedure described in Section 3.3.

The quantitative results of this comparison are obtained using the internal-validation procedure described above and are reported in Section 4.4.

### 3.6. Model Architectures

#### 3.6.1. Unimodal Deep Models

For the imaging and audio modalities, we use lightweight neural architectures designed for the small participant-level dataset.

The ANN+Attention model consists of a four-layer dense network (256–128–64–32 units, Leaky-ReLU activations, dropout =0.3) followed by an attention gate applied to the 32-dimensional representation before the classification head.

For text, the Text MLP consists of three fully connected layers (256–128–64 units) with Batch Normalization, ReLU activations, and dropout =0.4, followed by a classification head.

For the secondary fixed-split comparisons, Text MLP-1 and Text MLP-2 denote two hyperparameter configurations of this architecture. Text MLP-1 uses dropout =0.3, no learning-rate scheduler, and an early-stopping patience of 15 epochs, whereas Text MLP-2 uses dropout =0.4, ReduceLROnPlateau, and an early-stopping patience of 20 epochs.

The CNN+LSTM+Attention imaging model applies two Conv1D layers (64 and 128 filters, kernel size 3, padding 1), adaptive max pooling, a unidirectional LSTM with 64 hidden units, and an attention head before classification.

Because its input is the 148-dimensional mean-aggregated facial descriptor rather than a true temporal sequence, this architecture is not interpreted as a temporal model of facial behavior. The convolutional layers operate along the feature dimension, and the LSTM processes the resulting feature map as a pseudo-sequence. We therefore retain this architecture only as an auxiliary nonlinear sensitivity analysis against the simpler ANN+Attention model; modeling of genuine temporal facial dynamics would require frame-level or window-level sequences.

All deep models use AdamW with a learning rate of $10^{-3}$ and a weight decay of $10^{-4}$, class-weighted cross-entropy, gradient clipping with a maximum norm of 1.0, and early stopping. ReduceLROnPlateau uses a factor of 0.5 and a patience of 10 epochs where applicable.

Early-stopping and checkpoint selection are performed exclusively from the internal validation subset corresponding to each development partition and never from the held-out outer test fold.

#### 3.6.2. Early Fusion

Early fusion concatenates feature vectors from two or three modalities before classification. XGBoost [32] and LightGBM [33] classifiers are evaluated on the resulting joint feature spaces.

The tree-based configurations are kept deliberately low-complexity and fixed across the corresponding comparisons. XGBoost uses 200 estimators, maximum tree depth =4, learning rate =0.05, and scale_pos_weight =2.23; no subsampling parameter is explicitly specified, so the library default is retained. Its random state follows the script-level seed (SEED = 42 in fixed runs and the current paired experimental seed in seed-repeated analyses). LightGBM uses 200 estimators, num_leaves =31, learning rate =0.05, scale_pos_weight =2.23, and random seed =42; again, no subsampling parameter is explicitly specified, and the library default is retained. These settings are fixed before evaluation and are not tuned on the outer test folds.

#### 3.6.3. Trimodal Late Fusion

Late fusion combines modality-specific probability estimates rather than raw feature representations. The available unimodal models consist of Text MLP for text, ANN+Attention for audio, and ANN+Attention and CNN+LSTM+Attention for imaging.

Because two alternative imaging models are available, their probability outputs are first averaged to produce a single imaging-level probability,

$$p_{\mathrm{img}}=\frac{p_{\mathrm{ANN},\mathrm{img}}+p_{\mathrm{CNN}\text{-}\mathrm{LSTM},\mathrm{img}}}{2}.$$

The final trimodal probability is then obtained by assigning equal weight to the three modalities,

$$p_{\mathrm{trimodal}}=\frac{p_{\mathrm{text}}+p_{\mathrm{img}}+p_{\mathrm{audio}}}{3}.$$

This two-stage averaging prevents the imaging modality from receiving a larger implicit weight solely because two imaging architectures are evaluated. The two imaging estimators are treated as an architectural sensitivity ensemble within a single modality; their combination is not interpreted as evidence of temporal facial modeling.

The final classification threshold is selected exclusively from the corresponding internal-validation probabilities by maximizing Macro-F1 over t∈[0.30,0.75] with a step of 0.01. The selected threshold is subsequently applied unchanged to the held-out test predictions.

### 3.7. Explainability and Modality-Contribution Analysis

To directly analyze the two strongest trimodal models, we use grouped-modality permutation importance on trimodal LightGBM early fusion (the highest mean Macro-F1 model) and on trimodal late fusion (the second-highest mean Macro-F1 model). For each modality, all variables belonging to that modality are permuted jointly across held-out participants while the other modalities are left unchanged. For late fusion, the corresponding modality-level probability stream is permuted before the fixed fusion rule is applied. A total of 500 permutations are generated per modality. Importance is reported as the mean performance decrease relative to the unpermuted prediction, $\Delta F1=F1_{\mathrm{base}}-F1_{\mathrm{perm}}$ and $\Delta \mathrm{AUC}={\mathrm{AUC}}_{\mathrm{base}}-{\mathrm{AUC}}_{\mathrm{perm}}$. For Macro-F1, the decision threshold selected on the internal-validation subset is held fixed during all permutations and is never re-optimized on held-out labels.

Because grouped permutation can be difficult to interpret when modality blocks are statistically dependent, we additionally assess imaging–audio dependence using canonical correlation analysis (CCA) with a permutation-derived null distribution. The first canonical correlation is r=0.561 and exceeds the permutation-derived null threshold. This dependence check is used only to flag a potential source of attribution instability; it is not interpreted as evidence that correlation causally explains any difference between XAI rankings.

SHAP TreeExplainer [34] is applied to the XGBoost early-fusion model. For trimodal early fusion, SHAP values are computed over the 680-dimensional feature space, and mean absolute attribution values are aggregated by modality. For bimodal Text+Imaging early fusion, the corresponding analysis is performed on the 532-dimensional joint representation.

LIME [35] provides complementary local explanations by fitting an interpretable surrogate model in the neighborhood of individual predictions. Mean absolute LIME impact is aggregated by modality and class.

SHAP and LIME are retained as secondary diagnostics of the XGBoost models. They are not treated as modality weights for LightGBM or late fusion and are not directly equated with grouped-permutation importance because the methods explain different model families and quantify different notions of importance.

### 3.8. Computational Cost

To characterize computational requirements in addition to predictive performance, training time and inference latency are recorded for the principal models in CPU mode. Per-fit training time and per-participant inference latency are reported in Supplementary Table S9, together with the corresponding model family. These measurements provide an indication of the computational cost of the framework in a potential screening-oriented deployment setting.

### 3.9. Participant-Disjoint E-DAIC Generalization Protocol

To assess transfer beyond the primary 180-participant development cohort, we perform a participant-disjoint evaluation using E-DAIC [29].

#### 3.9.1. Overlap Control

E-DAIC contains Wizard-of-Oz sessions in the ID range 300–492 that overlap with DAIC-WOZ. Participants in this range are therefore excluded from the held-out generalization cohort. We retain only the 86 eligible participants with IDs 600–718, recorded using a fully autonomous AI-controlled interviewing agent and not represented during development.

Supplementary Table S1 provides the cohort-level reconciliation underlying this construction, including cohort size, class distribution, feature dimensionality, participant-ID range, and documented exclusions. This makes the distinction between the 180-participant primary cohort and the 189-participant E-DAIC-native development cohort explicit and auditable.

#### 3.9.2. Homogeneous Feature Re-Extraction

The original COVAREP and raw-landmark descriptors used in the primary DAIC-WOZ analysis are not available in identical form for the E-DAIC AI-agent sessions. We therefore express both the development and evaluation cohorts in a common E-DAIC-native feature space consisting of the following:

1. SBERT text embeddings (384 dimensions), using the same aggregation procedure as Section 3.2.1;
2. Mean-aggregated OpenFace 2.1.0 Action Unit, pose, and gaze descriptors (49 dimensions);
3. OpenSMILE eGeMAPS acoustic descriptors represented through participant- level means and standard deviations (46 dimensions).

This produces a homogeneous 479-dimensional representation.

The E-DAIC-native development cohort contains 189 of the 193 possible participant IDs in the range 300–492; IDs 342, 394, 398, and 460 are absent from the official E-DAIC archive.

For all E-DAIC analyses, binary depression status is defined consistently from the raw PHQ-8 score using the study threshold PHQ-8 ≥10. During participant-level cohort construction, raw PHQ-8 scores are cross-checked against the binary labels provided in the official E-DAIC files. When the two sources disagree, the PHQ-8-derived label is used to preserve a single outcome definition across all participants. This rule applies to 20 of the 275 pooled E-DAIC participants: 15 in the 189-participant development cohort and 5 in the 86-participant evaluation cohort. In all 20 cases, the raw PHQ-8 score is ≥10 while the corresponding official binary label indicates non-depression; no discordance in the opposite direction is observed. Using this consistent PHQ-8-based definition, the development cohort comprises 132 non-depressed and 57 depressed participants, whereas the participant-disjoint evaluation cohort comprises 57 non-depressed and 29 depressed participants. The complete E-DAIC pipeline was executed twice from independent kernel restarts and produced bit-for-bit identical outputs.

The relationship between this 189-participant cohort and the 180-participant primary DAIC-WOZ cohort is reported explicitly in Supplementary Table S1 rather than inferred from aggregate cohort counts.

#### 3.9.3. Model Training and Threshold Selection

Unimodal models are trained exclusively on the E-DAIC-native development cohort. Text uses the Text MLP, whereas the lower-dimensional imaging and audio representations use a lighter two-layer ANN+Attention model with 32 and 16 units.

Five-fold participant-level cross-validation within the development cohort produces out-of-fold predictions and five fitted models for each modality.

Within every development fold, checkpoint and early-stopping decisions are based only on an internal validation subset. Internal OOF reference metrics are computed from predictions generated for participants excluded from the corresponding model-fitting fold. No information from the 86 participant-disjoint E-DAIC evaluation subjects is used for feature selection, checkpoint selection, threshold optimization, calibration, or model fitting.

For each of the 86 evaluation participants, the modality-specific probability is obtained by averaging predictions from the five development-fold models before late fusion.

Any decision threshold used for the participant-disjoint evaluation is selected solely from predictions generated within the development cohort and is then applied unchanged to the 86 held-out E-DAIC participants.

#### 3.9.4. Verification of Unimodal Signal Independent of Architecture

To determine whether weak imaging or audio performance reflects the representation rather than a specific neural architecture, the E-DAIC-native imaging and audio descriptors are additionally evaluated using logistic regression, random forest, and gradient boosting under the same participant-level cross-validation protocol. Out-of-fold AUC is reported as a threshold-independent measure of discriminative ranking ability.

#### 3.9.5. Exploratory Alternative Late-Fusion Strategies

Given potential asymmetry in modality-level discriminative power, we additionally evaluate three alternatives to unweighted averaging: confidence-weighted fusion, text-guided gating, and stacked fusion.

For confidence-weighted fusion, modality weights are determined from development-set OOF AUC above chance level:

$$w_{m}=\frac{\max({\mathrm{AUC}}_{m}-0.5,0)}{\sum_{j}\max\left({\mathrm{AUC}}_{j}-0.5,0\right)}.$$

Text-guided gating assigns greater influence to the text prediction as its confidence increases, using its distance from 0.5, while allowing imaging and audio to contribute relatively more when the text prediction is uncertain.

Stacked fusion uses a logistic-regression meta-learner operating on the three modality-level probabilities.

For stacked fusion, meta-model fitting and evaluation are separated through an additional cross-validation level. Modality-level probabilities used to fit the logistic-regression combiner are generated exclusively from development participants, and the fitted meta-model is evaluated on participants whose probability outputs were not used to estimate the meta-model parameters. This nested construction prevents fitting and evaluating the fusion rule on the same OOF probability matrix.

These alternative fusion strategies are treated as exploratory analyses and are reported separately from the strictly held-out 86-participant E-DAIC generalization experiment.

## 4. Experiments and Results

### 4.1. Experimental Setup

Neural models were implemented in PyTorch (torch=2.0.1), whereas tree-based early-fusion models were implemented with XGBoost and LightGBM. All timing experiments reported here were executed in CPU mode. Macro-F1, defined as the unweighted mean of the class-specific F1 scores, was used as the primary performance metric because the dataset is imbalanced. Accuracy and ROC-AUC are additionally reported, together with sensitivity, specificity, positive predictive value (PPV), and negative predictive value (NPV) when clinically relevant operating characteristics are examined.

The primary evaluation is the participant-level 5-fold cross-validation protocol described in Section 3.3. At each fold, preprocessing, augmentation, model fitting, checkpoint selection, and decision-threshold optimization are confined to the corresponding development data.

In particular, the outer test fold is never used for feature scaling, augmentation, early-stopping decisions, checkpoint selection, threshold optimization, or hyperparameter selection. Reported test-fold metrics therefore correspond to models and thresholds that were fixed before the held-out participants were evaluated.

For cross-validation results, fold-level Macro-F1 is summarized as the mean ± standard deviation. In addition, because every participant appears in exactly one outer test fold, the five held-out prediction sets were concatenated to form one participant-level out-of-fold prediction vector (n=180). These pooled predictions were used for confusion-matrix-derived clinical metrics and participant-level paired comparisons.

For paired binary prediction comparisons, we use McNemar tests on participant-level held-out predictions, reporting the continuity-corrected $\chi^{2}$ statistic for the primary pooled comparison. McNemar’s test evaluates asymmetry in paired correctness outcomes and is not interpreted as a direct significance test of the Macro-F1 difference. Effect magnitude is therefore reported separately through absolute differences in Macro-F1, accuracy, sensitivity, specificity, PPV, and NPV. Given the limited sample size, statistical significance is not interpreted as the sole criterion of practical relevance.

The fixed 80/20 split (144/36 participants) is retained only as a secondary analysis for comparison with earlier fixed-split studies and for selected explainability analyses. Detailed fixed-split model results, bootstrap confidence intervals, and paired McNemar comparisons are reported in Supplementary Tables S2–S4.

Training time and per-participant inference latency are reported in Supplementary Table S9. In CPU mode, the neural models require seconds rather than minutes for an individual train/validation fit, whereas the complete five-fold multimodal experiment remains on the order of several minutes. These timings are intended to characterize computational requirements rather than to establish hardware-independent deployment benchmarks.

### 4.2. Primary 5-Fold Cross-Validation Results

Table 3 reports the primary participant-level 5-fold evaluation under the leakage-free protocol described in Section 3.3. The complete pipeline was reproduced bit-for-bit across two independent kernel restarts, including participant-level predictions, selected checkpoints, thresholds, and aggregate metrics.

LightGBM trimodal early fusion yields the highest mean Macro-F1 (0.542±0.025), while trimodal late fusion is second (0.532±0.041) and has the highest mean AUC among the trimodal models (0.558±0.054). The key controlled late-fusion comparison is Text+Imaging versus Text+Imaging+Audio: adding the audio branch increases the mean fold-level Macro-F1 from 0.446±0.021 to 0.532±0.041, a difference of +0.086. Text+Audio late fusion (0.481±0.055) also exceeds Text+Imaging late fusion by +0.035.

**Figure 2:**
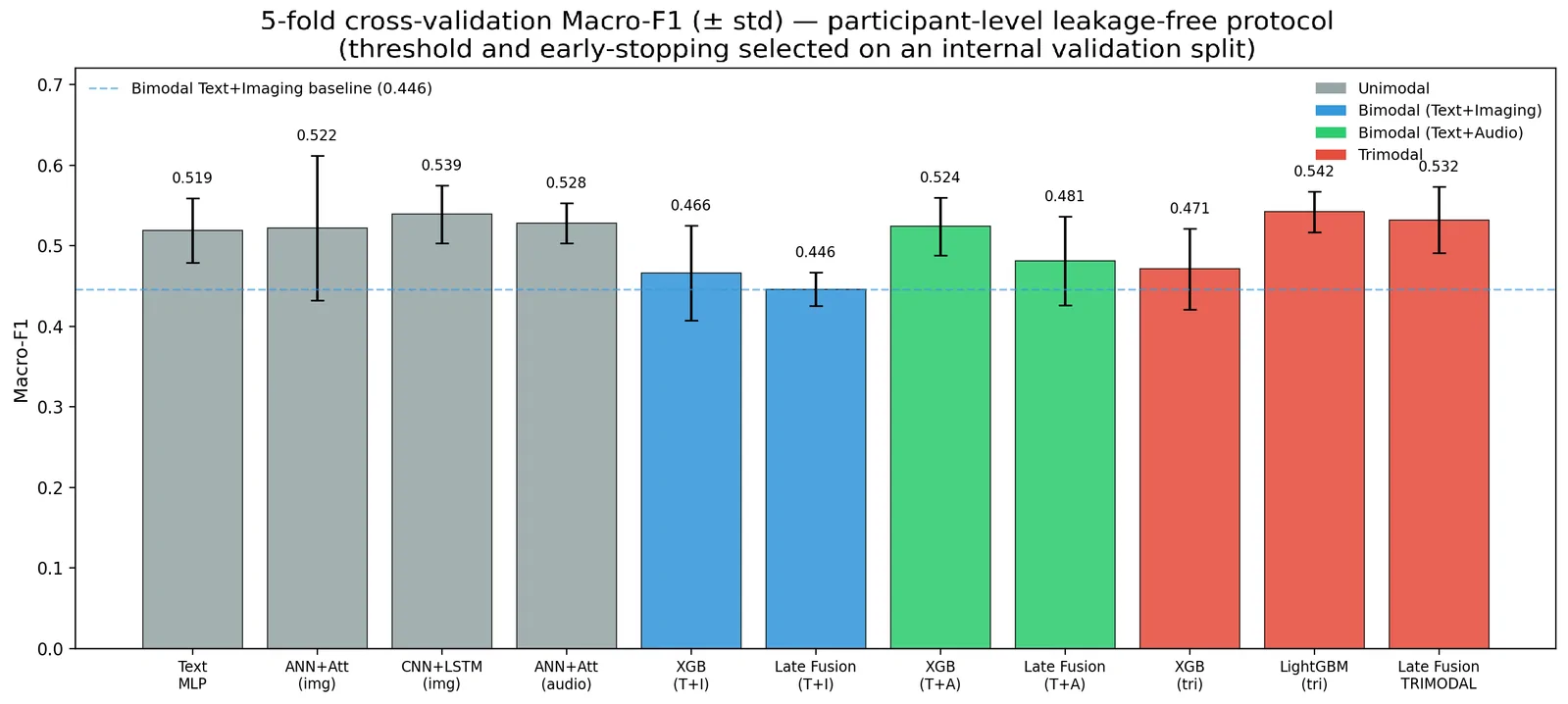
Mean 5-fold Macro-F1 with standard deviation under the participant-level leakage-free protocol. The highest mean Macro-F1 is obtained by trimodal LightGBM early fusion, followed by trimodal late fusion.

These results do not justify a universal claim that late fusion is superior to early fusion: the best mean Macro-F1 was obtained by trimodal LightGBM early fusion. Rather, the results show that the contribution of a modality depends jointly on representation and fusion rule. Figure 2 summarizes the mean 5-fold Macro-F1 performance of the evaluated configurations under the participant-level leakage-free protocol.

### 4.3. Participant-Level Statistical Comparison and Clinical Operating Characteristics

Because every participant contributes exactly one held-out prediction across the five outer folds, the pooled out-of-fold predictions (n=180) permit participant-level paired comparison without reducing the analysis to only five fold means.

For trimodal late fusion versus Text+Imaging late fusion, 81 participants are correctly classified by both models, 25 only by trimodal fusion, 9 only by Text+Imaging fusion, and 65 by neither. The continuity-corrected McNemar statistic is $\chi^{2}=6.618$ (p=0.010), and the exact two-sided McNemar test gives p=0.009. The complete participant-level correctness table underlying this paired comparison is reported in Supplementary Table S5. These tests concern paired classification correctness; the observed Macro-F1 differences are reported as effect magnitudes rather than assigned a McNemar-derived significance level. The discordant-pair ratio is 25/9≈2.78, meaning that, among participants for whom the two models disagree in correctness, trimodal fusion is correct approximately 2.8 times as often as Text+Imaging fusion. Trimodal fusion therefore produces 16 additional correct classifications, corresponding to an absolute accuracy increase of 8.9 percentage points on the pooled predictions. The pooled Macro-F1 increases from 0.465 to 0.537 (Δ=+0.072), consistent with the mean fold-level difference of +0.086. The corresponding pooled clinical operating characteristics are reported in Table 4. These effect magnitudes are reported alongside the paired test because statistical significance alone does not establish clinical importance, and the modest cohort size still warrants caution about precision and generalizability.

**Figure 3:**
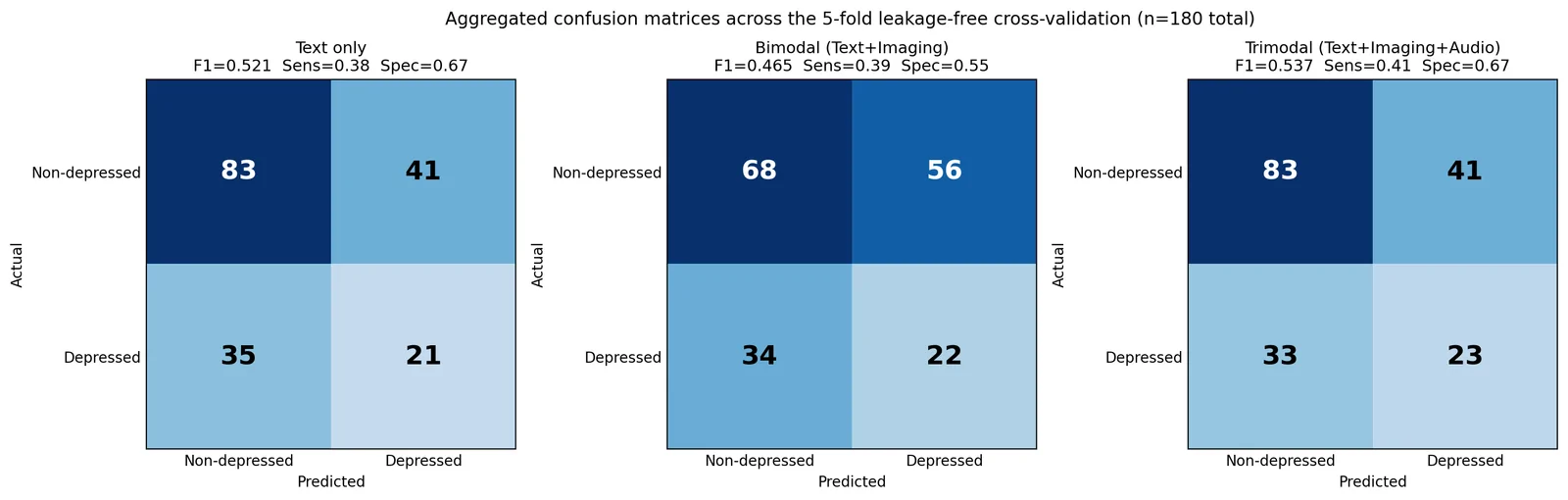
Aggregated confusion matrices for text-only, Text+Imaging late fusion, and trimodal late fusion, based on pooled held-out predictions across all five folds (n=180).

On the pooled predictions, trimodal late fusion has the highest Macro-F1, sensitivity, PPV, and NPV of the three configurations and ties text-only performance for the highest specificity. Relative to Text+Imaging fusion, the absolute changes are ΔSensitivity =+0.018, ΔSpecificity =+0.121, ΔPPV =+0.077, and ΔNPV =+0.049, while false negatives decrease from 34 to 33 and false positives from 56 to 41, as illustrated by the aggregated confusion matrices in Figure 3. The improvement is therefore not merely a sensitivity–specificity trade-off under the participant-level leakage-free protocol.

### 4.4. Controlled Augmentation-Leakage Analysis

The controlled leakage experiment compares the leakage-free augmentation procedure with a deliberately partition-violating MixUp construction under the same participant split, model architectures, hyperparameters, checkpoint-selection criterion, decision-threshold rule, and random-seed policy. In the leakage-free condition, the internal-validation subset is isolated before augmentation, and 250 synthetic samples are generated using parents drawn exclusively from the resulting sub-training partition. In the deliberately leaky condition, 250 MixUp candidates are generated from the full train+test participant pool, and a candidate is retained for development solely according to the partition membership of its first parent, while the second parent may belong to the held-out test set. Consequently, the realized number and composition of retained synthetic development samples are seed-dependent, and the internal-validation subset is subsequently drawn from this already augmented development pool. The comparison was repeated over five independent seeds for four fusion configurations and is interpreted as quantifying the net performance inflation associated with the partition-violating augmentation construction, rather than as a perfectly size-matched single-factor ablation. The resulting Macro-F1 values and performance inflation are reported in Table 5.

The deliberately leaky full-pool MixUp construction is associated with a Macro-F1 increase of 27.0–32.9 percentage points across the four configurations relative to the leakage-free comparator. For the bimodal late-fusion configuration, the mean difference is 30.1±9.5 percentage points. The paired conditions use the same participant split, architectures, hyperparameters, candidate augmentation budget, and seed policy, but the leaky construction can retain fewer than 250 synthetic development samples and draws internal validation only after the contaminated development pool has been formed. The observed difference should therefore be interpreted as the net inflation associated with the partition-violating augmentation construction, rather than as a pure causal effect of a single size-matched factor. This augmentation-related effect is distinct from model-selection leakage, which is prevented by restricting threshold and checkpoint selection to internal validation data.

### 4.5. Secondary Fixed-Split Analysis

The participant-level fixed 80/20 analysis uses 144 development participants and 36 held-out participants and is retained only for comparison with earlier fixed-split studies and selected XAI analyses.

For this secondary fixed-split analysis, LF-D denotes the late-fusion configuration combining Text MLP-2 with ANN+Attention imaging, with the imaging prediction receiving twice the weight of the text prediction. LF-F denotes the equally weighted model-level fusion of Text MLP-2, ANN+Attention imaging, and CNN+LSTM+Attention imaging. These secondary fixed-split configurations are distinct from the modality-balanced trimodal late-fusion strategy used in the primary 5-fold analysis.

Under the leakage-free fixed-split protocol, the best late-fusion fixed-split result is Accuracy =0.722, Macro-F1 =0.654, and AUC =0.655 (LF-F; LF-D has the same Accuracy and Macro-F1 and AUC =0.647). The strongest unimodal fixed-split configuration is CNN+LSTM imaging (Accuracy =0.722, Macro-F1 =0.699, and AUC =0.680). Because CNN+LSTM receives a single mean-aggregated 148-dimensional vector rather than a genuine temporal sequence, this observation is not interpreted as evidence of temporal-model superiority.

Fixed-split bootstrap intervals remain wide: for LF-F, Accuracy =0.722 [0.583–0.861] and Macro-F1 =0.654 [0.468–0.812]. McNemar comparisons are non-significant (LF-F vs. Text MLP-2: p=0.480; XGBoost early fusion vs. Text MLP-1: p=0.387; ANN+Attention vs. Text MLP-1: p=0.149). These analyses are therefore descriptive and are not used for the primary model-comparison claim. Detailed fixed-split tables are retained in the Supplementary Material to avoid fragmenting the main results.

### 4.6. Participant-Disjoint E-DAIC Evaluation

The E-DAIC analysis follows the protocol described in Section 3.9. Only the 86 participants with IDs 600–718 are used for held-out evaluation, thereby avoiding overlap with the DAIC-WOZ development participants.

Because E-DAIC belongs to the same corpus family, shares the PHQ-8 outcome, and has a related interviewing context, this experiment is explicitly interpreted as participant-disjoint within-family generalization rather than independent cross-corpus validation.

Both development and evaluation participants are represented using the same 479-dimensional E-DAIC-native feature space composed of SBERT transcript embeddings, OpenFace Action Unit/pose/gaze descriptors, and OpenSMILE eGeMAPS audio descriptors.

The 189-participant E-DAIC-native development cohort is distinct from the 180-participant primary DAIC-WOZ cohort because feature availability differs across the two analyses. Supplementary Table S1 reconciles the cohort definitions, class distributions, feature spaces, participant-ID ranges, and documented exclusions used in the two analyses.

#### 4.6.1. Internal E-DAIC-Native Reference

The internal reference is obtained from participant-level out-of-fold predictions on the E-DAIC-native development cohort.

Text remains the strongest individual branch in this homogeneous feature space (Macro-F1 =0.694±0.075), whereas the imaging and audio branches are substantially weaker. Trimodal unweighted averaging reaches 0.584±0.103 and, therefore, does not improve Macro-F1 over text alone. This pattern indicates that adding weak modality branches can dilute rather than reinforce the strongest modality (Table 6).

To determine whether weak non-text performance reflects the neural architecture or the feature representation, the imaging and audio descriptors are additionally evaluated using classical classifiers.

The classifier-specific AUC values are reported in Supplementary Table S10. Using the study-wide PHQ-8-based outcome definition, imaging AUC ranges from 0.396 to 0.530 and audio AUC from 0.526 to 0.575 across the three classical classifiers. These results indicate limited and classifier-dependent standalone discrimination in the non-text feature blocks, supporting the interpretation that their weakness is not specific to a single neural architecture.

#### 4.6.2. Held-Out E-DAIC Participants

Table 7 reports performance on the 86 participant-disjoint E-DAIC sessions using the study-wide PHQ-8-based outcome definition. Models and thresholds are determined entirely from the development cohort.

Using the study-wide PHQ-8-based outcome definition, the text-only branch provides the strongest overall held-out discrimination, with Macro-F1 =0.657 and AUC =0.710. Trimodal late fusion reaches Macro-F1 =0.621 and AUC =0.676. Relative to text alone, trimodal fusion has higher sensitivity (0.724 vs. 0.586) but lower specificity (0.579 vs. 0.737), indicating a sensitivity–specificity trade-off rather than a clear overall fusion advantage in this within-family evaluation.

We additionally compare the participant-level correctness of trimodal and text-only predictions on the 86 held-out E-DAIC participants. The complete discordance table contains 44 participants classified correctly by both models, 10 only by trimodal fusion, 15 only by text alone, and 17 by neither. The continuity-corrected McNemar statistic is $\chi^{2}=0.640$ (p=0.424), and the exact two-sided McNemar test likewise gives p=0.424. Thus, there is no evidence of a systematic difference in overall paired correctness between the two models.

These results support limited participant-disjoint transfer within the DAIC/E-DAIC corpus family, but they do not constitute evidence of independent cross-corpus clinical generalization.

### 4.7. Exploratory Adaptive Fusion

The E-DAIC-native results suggest that equal weighting can be suboptimal when modality-specific discriminative power is strongly asymmetric. We therefore examine confidence-weighted averaging, text-guided gating, and nested stacked fusion on the pooled E-DAIC-native sample.

Because this exploratory analysis pools the 189 development participants and 86 E-DAIC participants, it is not a held-out participant-disjoint generalization estimate. It is used only to compare combination rules within a common feature space.

Detailed values are reported in Supplementary Table S11. Using the study-wide PHQ-8-based outcome definition, unweighted averaging obtains AUC =0.650, compared with 0.686 for confidence-weighted fusion, 0.662 for text-guided gating, and 0.671 for nested stacked fusion.

All three adaptive strategies therefore remain above unweighted averaging in this exploratory pooled analysis, with confidence-weighted fusion providing the highest AUC. The result supports a feature-space-dependent view of fusion design: when modality quality is highly asymmetric, adaptive weighting can reduce dilution by weak branches. It does not establish independent cross-corpus generalization.

### 4.8. Explainability and Modality Contribution

Model-agnostic grouped permutation is used as the primary modality-contribution analysis because it can be applied directly to the two strongest trimodal models.

#### 4.8.1. Grouped-Modality Permutation Analysis

For LightGBM, text produces the largest mean Macro-F1 decrease (0.039), whereas imaging produces the largest AUC decrease (0.057). Audio has smaller mean decreases (0.016 Macro-F1 and 0.005 AUC). For late fusion, the mean decreases are more evenly distributed: text 0.031/0.021, imaging 0.014/0.023, and audio 0.020/0.013 for Macro-F1/AUC, respectively. Thus, the relative modality ranking depends on both the fitted model and the performance metric; these perturbation effects should not be interpreted as causal modality weights.

The LightGBM permutation ranking of imaging versus audio differs from the XGBoost SHAP profile reported below, where audio receives substantially greater mean absolute attribution than imaging. This is not a like-for-like contradiction: the analyses explain different fitted models and quantify different importance functionals. In addition, the imaging and audio blocks show non-negligible dependence (CCA r=0.561, above the permutation-derived null threshold), which can make marginal perturbation effects sensitive to shared information. We treat this dependence as a plausible interpretive factor, not as a demonstrated causal explanation of the ranking difference. The grouped-modality permutation results are summarized in Table 8.

#### 4.8.2. SHAP Diagnostic Analysis

SHAP TreeExplainer is applied to the trimodal XGBoost early-fusion model. The modality-level mean absolute attribution is 51.0% for text, 43.5% for audio, and 5.5% for imaging; the detailed values are reported in Supplementary Table S7. For comparison, the corresponding bimodal Text+Imaging SHAP attribution is reported in Supplementary Table S6.

**Figure 4:**
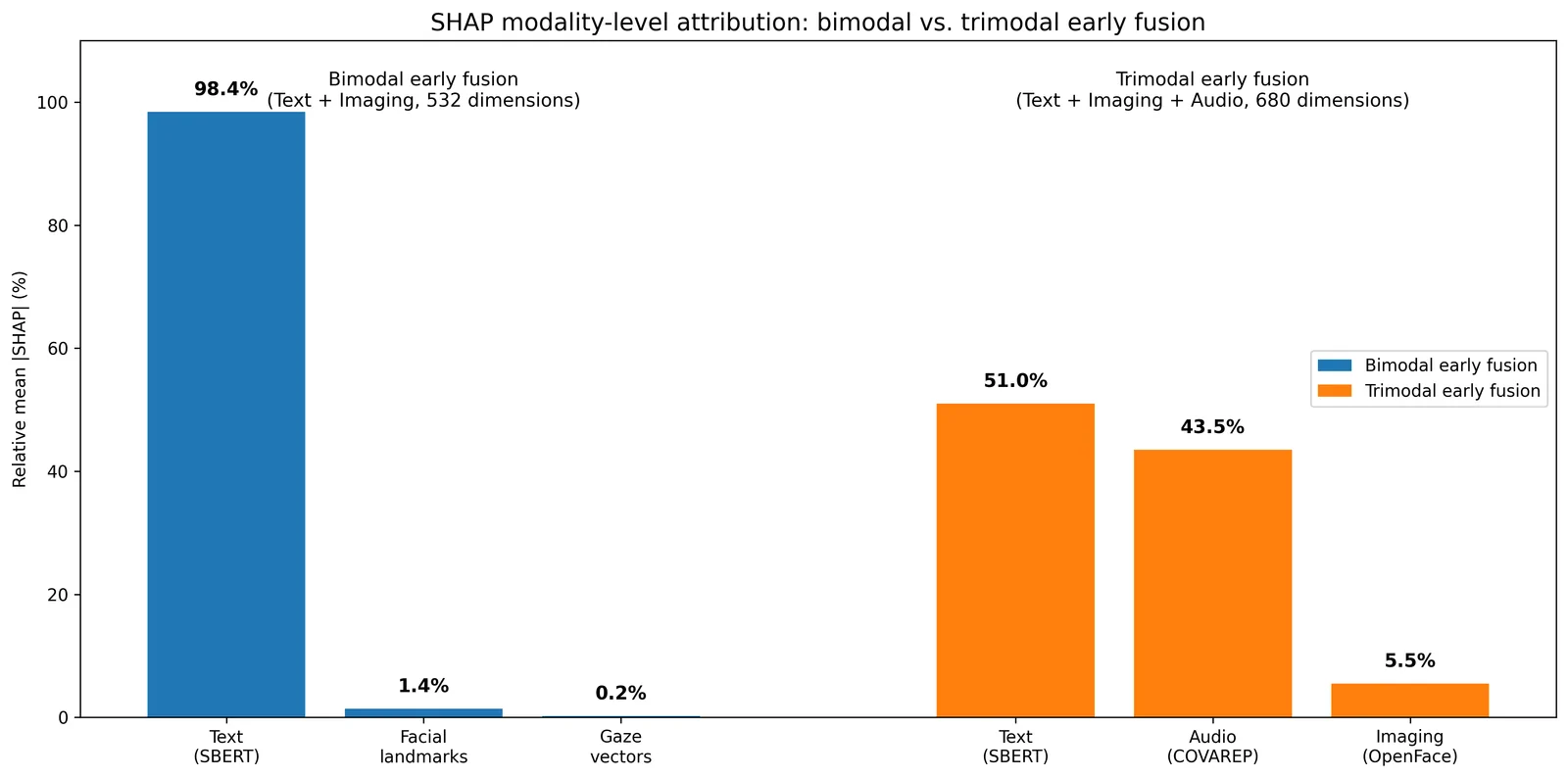
SHAP analysis of the trimodal XGBoost early-fusion model, with feature-level absolute attribution aggregated by modality.

The substantial COVAREP attribution shows that the XGBoost model uses acoustic features, but it should not be used to infer modality importance for LightGBM or late fusion. In particular, its audio-versus-imaging ranking differs from grouped permutation on LightGBM, reinforcing the need to interpret each XAI result with respect to the model and estimand it actually explains. The corresponding modality-level SHAP attribution is illustrated in Figure 4.

#### 4.8.3. LIME Analysis

LIME was evaluated on 30 participants from the fixed split. In the bimodal Text+Imaging model, mean absolute local impact is 0.916 for text, 0.276 for facial landmarks, and 0.020 for gaze, corresponding to an imaging/text impact ratio of 0.323. The ratio is 0.309 in non-depressed participants and 0.360 in depressed participants. Detailed class-specific and overall LIME results are reported in Supplementary Table S8. These values indicate that imaging can remain locally influential for selected predictions even when its global SHAP contribution is small.

The grouped-permutation analysis provides a direct model-agnostic perturbation analysis of both the highest-Macro-F1 LightGBM model and the trimodal late-fusion ensemble. LIME remains complementary local evidence from the XGBoost analysis and is not used to reconcile or override the grouped-permutation results.

## 5. Discussion

### 5.1. Evaluation Leakage and the Reliability of Reported Performance

The principal contribution of this study is methodological. Small participant-level behavioral datasets are particularly vulnerable to optimistic bias when observations derived from the same participant can influence both development and evaluation.

The paired experiment compares the leakage-free augmentation procedure with a deliberately partition-violating construction. In the leakage-free condition, both MixUp parents are restricted to the isolated post-split sub-training partition. In the deliberately leaky condition, parents are drawn from the full train+test pool, a synthetic sample is retained according to its first parent’s membership, the second parent may originate from the held-out test set, and internal validation is subsequently drawn from the resulting augmented development pool. Because 250 candidates are generated but only training-first-parent candidates are retained, the realized synthetic development-set size is not forced to match the leakage-free condition exactly. Across four configurations and five independent seeds, the leaky construction is nevertheless associated with a 27–33 percentage-point Macro-F1 increase; for Text+Imaging late fusion, the mean difference is 30.1±9.5 percentage points (0.822±0.079 vs. 0.521±0.075). We therefore interpret this as the net inflation associated with the partition-violating augmentation construction, not as a perfectly size-matched causal ablation.

A distinct source of model-selection leakage arises when held-out test labels are used to select decision thresholds or neural checkpoints. This can bias threshold-dependent performance even when participants remain physically separated across development and evaluation sets. In the protocol used here, these choices are confined to an internal validation split. Deterministic participant ordering and per-model seed resetting further support reproducibility, and the primary pipeline produced bit-for-bit identical outputs across two independent full-kernel restarts.

These findings reinforce the need to report not only participant-level splitting but also the scope of augmentation parent sampling and synthetic-sample assignment, preprocessing scope, early-stopping criteria, threshold selection, and randomization policy. The resulting performance estimates should therefore be interpreted as a leakage-audited reference rather than as a leaderboard-oriented claim.

### 5.2. What Does Multimodal Fusion Add?

Under the participant-level leakage-free protocol, trimodal late fusion achieves Macro-F1 =0.532±0.041, compared with 0.446±0.021 for Text+Imaging late fusion, a mean difference of +0.086. The pooled participant-level comparison favors trimodal fusion ($\chi^{2}=6.618$, p=0.010), with 25 participants correctly classified only by trimodal fusion versus 9 only by the bimodal model.

The evidence is consistent with a useful acoustic contribution. Text+Audio late fusion (0.481±0.055) exceeds Text+Imaging late fusion (0.446±0.021), and the two highest mean Macro-F1 values in the full model comparison are obtained by trimodal LightGBM early fusion (0.542±0.025) and trimodal late fusion (0.532±0.041). In the early-fusion SHAP analysis, COVAREP accounts for 43.5% of global attribution, compared with 51.0% for text and 5.5% for imaging. Together, these results are consistent with complementarity of the acoustic representation, although SHAP should not be read as a direct explanation of the neural late-fusion output.

The pooled clinical metrics also support this interpretation without requiring a sensitivity–specificity trade-off: trimodal late fusion reaches sensitivity =0.411 and specificity =0.669, compared with 0.393 and 0.548 for Text+Imaging fusion. It has the highest Macro-F1, sensitivity, PPV, and NPV among the three pooled configurations and ties text alone for specificity. The absolute sensitivity remains modest, however, so these results do not support a clinical-readiness claim.

### 5.3. Fusion Strategy Depends on Modality Quality

The experiments do not support a universal preference for early or late fusion. On the primary DAIC-WOZ representation, trimodal LightGBM early fusion has the highest mean Macro-F1 (0.542±0.025), only slightly above trimodal late fusion (0.532±0.041). On the lower-dimensional E-DAIC-native representation, text is much stronger than the non-text branches and unweighted trimodal averaging does not exceed text-only Macro-F1. In the exploratory pooled E-DAIC-native analysis using the study-wide PHQ-8-based outcome definition, confidence weighting, text-guided gating, and nested stacking obtain AUC values of 0.686, 0.662, and 0.671, respectively, compared with 0.650 for uniform averaging.

This pattern indicates that fusion should be adapted to empirically measured modality quality rather than chosen a priori. Richer COVAREP descriptors can provide useful complementary signal in the primary feature space, whereas weaker E-DAIC-native non-text representations can dilute an unweighted ensemble.

### 5.4. Interpretation of Participant-Disjoint E-DAIC Generalization

The 86-participant E-DAIC evaluation is deliberately described as within-family generalization. DAIC-WOZ and E-DAIC share the PHQ-8 outcome, related interview design, and corpus provenance; therefore, the experiment is not equivalent to validation on an independently collected corpus.

Within this scope, using the study-wide PHQ-8-based outcome definition, text alone reaches Macro-F1 =0.657 and AUC =0.710, compared with Macro-F1 =0.621 and AUC =0.676 for trimodal late fusion. Thus, trimodal fusion does not improve overall discrimination relative to the text-only branch in the E-DAIC-native evaluation. Its practical difference lies instead in the operating characteristics: sensitivity increases from 0.586 for text alone to 0.724 for trimodal fusion, while specificity decreases from 0.737 to 0.579. The paired McNemar comparison is non-significant (p=0.424). This trade-off may be relevant in screening settings that prioritize sensitivity, but its clinical utility cannot be established from this retrospective analysis and should not be interpreted as a general superiority claim.

### 5.5. Explainability: Scope and Interpretation

The XAI results should be interpreted according to the model they actually explain. Grouped permutation directly evaluates modality contribution in the two strongest trimodal models. For LightGBM, text causes the largest Macro-F1 decrease (0.039) while imaging causes the largest AUC decrease (0.057); for late fusion, the three modalities produce smaller and more balanced decreases. The XGBoost SHAP profile (51.0% text, 43.5% audio, 5.5% imaging) therefore should not be used as a surrogate explanation of LightGBM or late fusion. The difference between the LightGBM permutation ranking and the XGBoost SHAP ranking is scientifically informative but not directly attributable to a single cause: the analyses concern different fitted models, use different importance definitions, and the imaging and audio representations are themselves dependent (CCA r=0.561 above the permutation-derived null threshold). We therefore report the divergence rather than forcing an artificial reconciliation.

### 5.6. Limitations

Several limitations constrain the conclusions. First, the primary cohort contains only 180 participants, including 56 depressed participants, so fold-level estimates remain variable and pairwise effect estimates should be interpreted cautiously even when the pooled McNemar test indicates asymmetric paired correctness. Repeated or nested cross-validation on a larger cohort would provide more stable uncertainty estimates. In addition, SBERT was fixed after a preliminary non-nested representation comparison on the same overall cohort; the primary results are therefore conditional on that representation choice rather than evidence from a fully nested encoder-selection procedure.

Second, the visual and acoustic features are aggregated at session level. In particular, the CNN+LSTM imaging model receives a mean-aggregated 148-dimensional vector rather than a true temporal sequence; its performance should therefore not be interpreted as evidence about temporal dynamics.

Third, the E-DAIC evaluation is participant-disjoint but not independent cross-corpus validation. A stronger generalization test would use a separately collected clinical dataset and, ideally, matched feature extraction across cohorts.

Fourth, the grouped-permutation analysis is model-agnostic and is applied directly to LightGBM and late fusion, but it remains an associational perturbation analysis rather than a causal decomposition. Correlated modality blocks can redistribute apparent importance, and SHAP on XGBoost is not directly comparable with permutation importance on LightGBM. Conditional permutation, interaction-aware attribution, and replication on larger cohorts would be needed for stronger modality-specific causal interpretations.

Finally, this study is a retrospective computational proof of concept. Prospective validation, fairness analysis, calibration assessment, clinical utility evaluation, and regulatory considerations would be required before any deployment claim.

### 5.7. Future Directions

Future work should prioritize three extensions. First, re-extracting comparable high-fidelity acoustic and facial descriptors from E-DAIC would help separate dataset shift from feature-space degradation. Second, temporally aware models should replace session-level averaging where raw sequential signals are available. Third, adaptive or learned fusion should be tested on the original COVAREP/landmark feature space under the same nested, leakage-free protocol used here.

Future XAI work should extend the present grouped-permutation analysis with conditional or interaction-aware perturbation methods that explicitly account for dependence between modality blocks. Validation on an independently collected corpus also remains necessary before stronger claims about modality causality or clinical generalization can be made.

## 6. Conclusions

This study presents a leakage-audited trimodal depression-screening framework on DAIC-WOZ and shows that evaluation design can materially alter both absolute performance and model ranking. Under a controlled five-seed comparison, a deliberately leaky full-pool MixUp construction that can incorporate a held-out participant as the second parent of a development synthetic sample inflates Macro-F1 by 27–33 percentage points across four fusion configurations. The evaluation protocol also prevents model-selection leakage by restricting decision-threshold and neural-checkpoint selection to internal validation data.

Under the leakage-free and reproducibility-controlled 5-fold protocol, trimodal late fusion improves mean Macro-F1 from 0.446±0.021 for Text+Imaging fusion to 0.532±0.041; the pooled participant-level paired-correctness comparison also favors trimodal fusion (McNemar p=0.010). Using the study-wide PHQ-8-based outcome definition, the 86-participant within-family evaluation yields Macro-F1 =0.621 and AUC =0.676 for trimodal late fusion. This experiment remains interpreted as participant-disjoint within-family generalization rather than independent cross-corpus validation.

Overall, the results support a cautious, protocol-dependent view of multimodal complementarity. Reliable claims require participant-level separation before augmentation, development-only model selection, transparent metric definitions, reproducible randomization procedures, and validation on genuinely independent cohorts before clinical deployment can be considered.

## Figures and Tables

**Table 1:** Representative studies in depression detection using DAIC-WOZ, E-DAIC, or related clinical-interview corpora. Performance values retain the metric definition reported in the original source; an unspecified F1 definition is not assumed to be equivalent to Macro-F1. Consequently, absolute values across rows should not be interpreted as directly comparable.

Study	Modalities	Dataset	Reported Metric	Metric Definition	Evaluation
Flores et al. [15]	Facial (eye gaze)	DAIC-WOZ	0.69 average; 0.81 best question-specific	Binary positive-class F1	Fixed 80/20 split; 0.81 is the best D7 question-specific result
DAAM [18]	Audio	DAIC-WOZ	F1 = 0.720	Macro-F1	Fixed evaluation
Zhang et al. [22]	Audio+Video+Text	Custom clinical cohort	0.76 interview-only; 0.78 reading+interview	Binary positive-class F1	Balanced 240-subject cohort; stratified 70/30 holdout; 0.78 includes task-level decision fusion
Nykoniuk et al. [23]	Audio+Text	DAIC-WOZ+E-DAIC	0.79 (source-reported test F1)	Positive-class F1 in source text	Single 80/20 held-out split; modified 180-participant cohort; early vs. late representation fusion
Sadeghi et al. [28]	Text+Visual	E-DAIC	MAE = 2.85	Regression	Development set
Ishikawa & Duke [24]	Text+Audio (+LLM score)	E-DAIC	F1 = 0.723	Macro-F1	Subject-disjoint LOSO-CV
This work (bimodal)	Text+Imaging	DAIC-WOZ	F1 = 0.446±0.021	Macro-F1	5-fold participant-level CV
This work (trimodal)	Text+Imaging+Audio	DAIC-WOZ	F1 = 0.532±0.041	Macro-F1	5-fold participant-level CV
This work (within-family)	Text+Imaging+Audio	E-DAIC (n=86)	F1 = 0.621	Macro-F1	Participant-disjoint holdout

**Table 2:** Preliminary embedding comparison using identical 3-fold cross-validation partitions and a common held-out evaluation set. SBERT (all-MiniLM-L6-v2) provides the highest held-out Macro-F1 while retaining a compact 384-dimensional representation.

Embedding	Dim	3-Fold CV Macro-F1	Held-Out Macro-F1
SBERT (all-MiniLM-L6-v2)	384	0.563	0.679
PubMedBERT	768	0.587	0.457
BioBERT	768	0.593	0.500
ClinicalBERT	768	0.538	0.429
RoBERTa	768	0.617	0.563

**Table 3:** Participant-level 5-fold cross-validation results on DAIC-WOZ (n=180). Mean ± standard deviation over five outer folds. All preprocessing, model selection, checkpoint selection, and decision-threshold optimization are confined to the corresponding development data.

Configuration	Model	Accuracy	Macro-F1	AUC
Unimodal	Text MLP (SBERT)	0.578±0.057	0.519±0.040	0.567±0.029
	ANN+Attention (imaging)	0.572±0.109	0.522±0.090	0.568±0.055
	CNN+LSTM (imaging)	0.589±0.037	0.539±0.036	0.558±0.058
	ANN+Attention (audio)	0.617±0.064	0.528±0.025	0.526±0.074
Text+Imaging	XGBoost early fusion	0.544±0.089	0.466±0.059	0.543±0.118
	Late fusion	0.500±0.063	0.446±0.021	0.552±0.029
Text+Audio	XGBoost early fusion	0.606±0.044	0.524±0.036	0.507±0.091
	Late fusion	0.606±0.064	0.481±0.055	0.562±0.054
Trimodal	XGBoost early fusion	0.567±0.084	0.471±0.050	0.528±0.111
	LightGBM early fusion	0.656±0.014	0.542±0.025	0.547±0.090
	Late fusion	0.589±0.059	0.532±0.041	0.558±0.054

**Table 4:** Clinical operating characteristics obtained by pooling participant-level held-out predictions across the five outer folds (n=180).

Model	Macro-F1	Sens.	Spec.	PPV	NPV	TN/FP/FN/TP
Text MLP	0.521	0.375	0.669	0.339	0.703	83/41/35/21
Text+Imaging late fusion	0.465	0.393	0.548	0.282	0.667	68/56/34/22
Trimodal late fusion	0.537	0.411	0.669	0.359	0.716	83/41/33/23

**Table 5:** Macro-F1 under deliberately leaky full-pool versus leakage-free post-split MixUp construction. Mean ± standard deviation over five paired independent seeds on fixed 80/20 splits. Within each seed, the leaky and leakage-free conditions use the same participant partition. Inflation is defined as leaky minus leakage-free Macro-F1.

Configuration	Leaky Macro-F1	Leakage-Free Macro-F1	Inflation
Bimodal late fusion (Text+Imaging)	0.822±0.079	0.521±0.075	+0.301±0.095
Bimodal XGBoost early fusion	0.795±0.060	0.500±0.068	+0.295±0.037
Trimodal late fusion	0.785±0.054	0.514±0.090	+0.270±0.064
Trimodal XGBoost early fusion	0.803±0.083	0.474±0.082	+0.329±0.078

**Table 6:** Internal out-of-fold 5-fold cross-validation results on the 189-participant E-DAIC-native development cohort using SBERT (384 dimensions), OpenFace AU/pose/gaze (49 dimensions), and OpenSMILE eGeMAPS (46 dimensions). Depression status is defined from the raw PHQ-8 score using the study-wide threshold PHQ-8 ≥10. The development cohort comprises 132 non-depressed and 57 depressed participants. Values are mean ± standard deviation across folds.

Modality	Macro-F1
Text (SBERT, 384 dimensions)	0.694±0.075
Imaging (OpenFace-AU, 49 dimensions)	0.397±0.077
Audio (eGeMAPS, 46 dimensions)	0.421±0.045
Trimodal late fusion	0.584±0.103

**Table 7:** Participant-disjoint within-family evaluation on 86 E-DAIC participants (IDs 600–718; no overlap with development). Depression status is defined from the raw PHQ-8 score using the study-wide threshold PHQ-8 ≥10. The evaluation cohort comprises 57 non-depressed and 29 depressed participants. Results were reproduced bit-for-bit across two independent kernel restarts.

Modality	Acc	Macro-F1	AUC	Sens.	Spec.
Text (SBERT, 384 dimensions)	0.686	0.657	0.710	0.586	0.737
Imaging (OpenFace-AU, 49 dimensions)	0.570	0.530	0.515	0.414	0.649
Audio (eGeMAPS, 46 dimensions)	0.500	0.500	0.569	0.759	0.368
Trimodal late fusion	0.628	0.621	0.676	0.724	0.579

**Table 8:** Grouped-modality permutation importance for the two strongest trimodal models. Values are mean performance decreases over 500 permutations per modality. The Macro-F1 decision threshold remains fixed at the value selected on internal validation.

Model	Permuted Modality	ΔMacro-F1	ΔAUC
LightGBM early fusion	Text	0.039	0.006
	Imaging	0.035	0.057
	Audio	0.016	0.005
Trimodal late fusion	Text	0.031	0.021
	Imaging	0.014	0.023
	Audio	0.020	0.013

## Data Availability

The DAIC-WOZ and E-DAIC datasets are available under the access conditions established by the USC Institute for Creative Technologies. The analysis code corresponding to the results reported in this manuscript is publicly available in the companion GitHub repository: https://github.com/Nesma876/Multimodal-Depression-Screening-Text-Facial-Behavior-and-Prosodic-Fusion (accessed on 18 August 2026). The repository documents the participant-level cohort construction, leakage-free and deliberately partition-violating augmentation procedures, primary 5-fold and fixed-split training workflows, development-only checkpoint and threshold selection, participant-level statistical analyses, E-DAIC within-family evaluation, and explainability/modality-contribution analyses. Raw DAIC-WOZ/E-DAIC interview media, transcripts, and restricted derived feature files are not redistributed because they remain subject to the USC ICT data-use conditions. Authorized corpus users can use the documented input schemas and processing scripts to reconstruct the required analysis inputs in their approved data environment.

## Supplementary Materials

The following supporting information can be downloaded at https://www.mdpi.com/article/10.3390/bioengineering13091009/s1, Table S1: Reconciliation of the participant cohorts used in the primary DAIC-WOZ analysis and the E-DAIC-native generalization analysis. Depression is defined using PHQ-8 ≥10 throughout. Table S2: Model performance on the leakage-free fixed 80/20 split ($n_{\mathrm{test}}=36$). Threshold and neural checkpoint selection are restricted to the internal validation subset. Table S3: Bootstrap 95% confidence intervals based on 1000 resamples of the fixed held-out set (n=36). Table S4: Complete paired-correctness counts and McNemar tests on the leakage-free fixed split (n=36). $\chi_{\mathrm{cc}}^{2}$ denotes the continuity-corrected McNemar statistic. The exact *p*-value is from the two-sided binomial form of McNemar’s test. Table S5: Complete participant-level correctness table for the primary comparison between trimodal late fusion and Text+Imaging late fusion, based on pooled out-of-fold predictions from all five folds (n=180). Table S6: Modality-level SHAP attribution for the bimodal Text+Imaging XGBoost early-fusion model. Table S7: Modality-level SHAP attribution for the trimodal Text+Imaging+Audio XGBoost early-fusion model on the leakage-free fixed split. Table S8: Mean absolute LIME impact by modality for the bimodal XGBoost early-fusion model. Table S9: Observed computational-cost ranges for the principal model families in the participant-level leakage-free pipeline. Training time denotes one train/validation fit; inference latency is reported per participant. Table S10: Supplementary out-of-fold AUC of E-DAIC-native imaging and audio descriptors using classical classifiers under the study-wide PHQ-8-based outcome definition. Values around 0.5 indicate limited standalone ranking ability. Table S11: Supplementary exploratory AUC comparison of fusion rules on the pooled E-DAIC-native feature space using the study-wide PHQ-8-based outcome definition (PHQ-8 ≥10).

## Abbreviations

ANN	Artificial Neural Network
AUC	Area Under the Receiver Operating Characteristic Curve
BN	Batch Normalization
COVAREP	Collaborative Voice Analysis Repository for Speech Technologies
CV	Cross-Validation
DAIC-WOZ	Distress Analysis Interview Corpus–Wizard of Oz
EF	Early Fusion
F0	Fundamental Frequency
LF	Late Fusion
LightGBM	Light Gradient Boosting Machine
LIME	Local Interpretable Model-Agnostic Explanations
MDD	Major Depressive Disorder
MLP	Multilayer Perceptron
NPV	Negative Predictive Value
PHQ-8	Patient Health Questionnaire-8
PPV	Positive Predictive Value
ROC	Receiver Operating Characteristic
SBERT	Sentence-BERT
SHAP	SHapley Additive exPlanations
UAR	Unweighted Average Recall
XAI	Explainable Artificial Intelligence
XGBoost	Extreme Gradient Boosting
