# Supplementary material for: Leakage-Free Multimodal Depression Screening: Controlled Evaluation of Text, Facial Behavior, and Prosodic Fusion
Source: Bioengineering (Basel). 2026 Aug 30;13(9):1009. doi: 10.3390/bioengineering13091009 (PMC13603094; doi:10.3390/bioengineering13091009)
Supplement: Supplementary file 1 [file bioengineering-13-01009-s001.zip › bioengineering-4472594-Supplementary.pdf]

bioengineering

MDPI

Article

Leakage-Free Multimodal Depression Screening: Controlled Evaluation of Text, Facial Behavior, and Prosodic Fusion

Souaad Hamza-Cherif [1,*] and Nesma Settouti [2]

[1] Biomedical Engineering Laboratory, University of Tlemcen, 13000 Tlemcen, Algeria
[2] LabISEN, LSL, ISEN Ouest, 14000 Caen, France
* Correspondence: souad.hamzacherif@univ-tlemcen.dz

Supplementary Materials

The supplementary material provides additional information supporting the cohort audit, secondary fixed-split analyses, paired statistical comparisons, and post-hoc explainability analyses reported in the revised manuscript. The primary scientific conclusions remain based on the participant-level 5-fold evaluation reported in the main text.

S1. Cohort Reconciliation and Generalization Audit

The primary DAIC-WOZ analysis and the E-DAIC-native generalization analysis use different feature requirements and therefore involve overlapping but non-identical development cohorts. Table S1 summarizes the independently audited cohort definitions used throughout the revised manuscript.

Table S1. Reconciliation of the participant cohorts used in the primary DAIC-WOZ analysis and the E-DAIC-native generalization analysis. Depression is defined using PHQ-8 $\geq$ 10 throughout.

Cohort	n	Non-Depressed	Depressed	Feature Dim.	Definition/Audit Information
Primary DAIC-WOZ cohort	180	124	56	680	SBERT (384d) + OpenFace landmark/gaze (148d) + COVAREP (148d); participants retained after modality-specific quality control.
E-DAIC-native development cohort	189	132	57	479	IDs in the 300–492 range represented using homogeneous SBERT + OpenFace AU/pose/gaze + eGeMAPS features. IDs 342, 394, 398, and 460 are absent from the official E-DAIC archive. Fifteen labels were corrected from non-depressed to depressed after cross-checking raw PHQ-8 scores ($\geq$ 10) against the official label files.
Participant-disjoint E-DAIC evaluation cohort	86	57	29	479	Eligible sessions with IDs 600–718; no participant overlap with the E-DAIC-native development cohort. Five labels were corrected from non-depressed to depressed after the PHQ-8 audit.
Combined E-DAIC-native feature file	275	189	86	479	189 development + 86 held-out participants; independently verified to contain no duplicated participant IDs and no missing feature values. Across the pooled file, 20/275 labels were corrected from raw PHQ-8 scores; no reverse discordance was observed.

The 189-participant E-DAIC-native development cohort should therefore not be interpreted as the 180-participant primary DAIC-WOZ cohort with four participants removed. The two cohorts are reconstructed independently because their required imaging and acoustic representations differ. In addition, the participant-level audit identified a systematic inconsistency between the official E-DAIC binary labels and the raw PHQ-8 scores for 20/275 pooled participants. Because depression is defined as PHQ-8 ≥ 10 throughout this study, those 20 labels were recomputed from the raw score before the final E-DAIC analyses.

S2. Secondary Fixed-Split Results

The fixed 80/20 analysis uses 144 development participants and 36 held-out participants. These results are included for transparency and comparison with earlier fixed-split analyses but are not used as the primary evidence for model superiority.

Table S2. Regenerated model performance on the leakage-free fixed 80/20 split ($n_{\text{test}} = 36$). Threshold and neural checkpoint selection are restricted to the internal validation subset.

Type	Model	Accuracy	Macro-F1	AUC
Text	Text MLP-1	0.694	0.649	0.615
Text	Text MLP-2	0.667	0.556	0.658
Imaging	ANN+Attention	0.528	0.392	0.520
Imaging	CNN+LSTM+Attention	0.722	0.699	0.680
Early fusion	XGBoost Text+Imaging	0.583	0.540	0.705
Late fusion	LF-D	0.722	0.654	0.647
Late fusion	LF-F	0.722	0.654	0.655

LF-D combines Text MLP-2 and ANN+Attention imaging, with the imaging prediction weighted twice relative to the text prediction. LF-F combines Text MLP-2, ANN+Attention imaging, and CNN+LSTM+Attention imaging with equal model-level weights. These fixed-split configurations are distinct from the modality-balanced trimodal late-fusion architecture used in the primary 5-fold evaluation.

S2.1. Bootstrap Con idence Intervals

The wide and substantially overlapping intervals illustrate the limited precision of performance estimates obtained from a 36-participant fixed test set.

Table S3. Bootstrap 95% confidence intervals based on 1,000 resamples of the fixed held-out set ($n = 36$).

Model	Accuracy [95% CI]	Macro-F1 [95% CI]	AUC
Text MLP-2	0.667 [0.500–0.806]	0.556 [0.379–0.730]	0.658
ANN+Attention (imaging)	0.528 [0.361–0.694]	0.392 [0.278–0.535]	0.520
XGBoost early fusion	0.583 [0.417–0.750]	0.540 [0.377–0.699]	0.705
LF-F	0.722 [0.583–0.861]	0.654 [0.468–0.812]	0.655

S3. Paired Participant-Level Statistical Comparisons

S3.1. McNemar Tests on the Fixed Split

Table S4 reports the complete paired-correctness counts underlying the regenerated fixed-split McNemar comparisons. “A only” denotes participants classified correctly by Model A but incorrectly by Model B; “B only” denotes the converse.

For LF-F versus Text MLP-2, the corresponding continuity-corrected statistic is $\chi^2_{cc} = 0.500$. For XGBoost early fusion versus Text MLP-1 it is $\chi^2_{cc} = 0.750$, and for

ANN+Attention versus Text MLP-1 it is $\chi^2_{cc} = 2.083$. None of these fixed-split comparisons is statistically significant.

Table S4. Complete paired-correctness counts and McNemar tests on the regenerated fixed split ($n = 36$). χ^2_{cc} denotes the continuity-corrected McNemar statistic. The exact p-value is from the two-sided binomial form of McNemar's test.

Model A	Model B	Both Correct	A Only	B Only	Both Wrong	p_{cc}	p_{exact}
LF-F	Text MLP-2	24	2	0	10	0.480	0.500
XGBoost early fusion	Text MLP-1	17	4	8	7	0.387	0.388
ANN+Attention	Text MLP-1	16	3	9	8	0.149	0.146

S3.2. Primary Pooled McNemar Comparison

Unlike the secondary fixed-split tests, the primary comparison uses one held-out out-of-fold prediction for every participant in the 5-fold evaluation ($n = 180$).

Table S5. Complete participant-level correctness table for the primary comparison between trimodal late fusion and Text+Imaging late fusion, based on pooled out-of-fold predictions from all five folds ($n = 180$).

	**Text+Imaging correct**	**Text+Imaging incorrect**
**Trimodal correct**	81	25
**Trimodal incorrect**	9	65

The discordant counts are therefore 25 and 9. The continuity-corrected McNemar test gives

$$\chi^2_{cc} = 6.618, \qquad p = 0.010,$$

and the exact two-sided McNemar test gives

$$p_{exact} = 0.009.$$

Thus, trimodal fusion produces 16 additional correct participant-level classifications relative to Text+Imaging fusion, corresponding to an absolute accuracy increase of 8.9 percentage points on the pooled out-of-fold predictions. This participant-level comparison is the primary statistical evidence supporting the trimodal late-fusion advantage.

S4. Supplementary Explainability Analyses

SHAP and LIME are applied to XGBoost early-fusion models rather than directly to the final neural soft-voting late-fusion ensemble. They should therefore be interpreted as diagnostic analyses of modality-specific information in the feature space and not as direct decompositions of the final late-fusion predictions.

S4.1. Bimodal SHAP Analysis

Table S6. Re-verified modality-level SHAP attribution for the bimodal Text+Imaging XGBoost early-fusion model.

**Modality**	**Number of Features**	**Relative Mean |SHAP|**
Text (SBERT)	384	**98.4%**
Facial landmarks	136	1.4%
Gaze vectors	12	0.2%
**Total**	532	100%

The bimodal XGBoost model is therefore dominated by the SBERT representation, with facial-behavior descriptors accounting for only 1.6% of global SHAP importance. This result characterizes the bimodal early-fusion model only and should not be interpreted as a modality weighting of the late-fusion ensemble.

S4.2. Trimodal SHAP Analysis

Adding COVAREP audio substantially changes the early-fusion attribution profile. Audio accounts for 43.5% of global SHAP importance, compared with 51.0% for text and 5.5% for imaging. This supports the presence of substantial acoustic information in the trimodal feature space. It does not, however, establish modality weights for the final late-fusion classifier.

Table S7. Re-verified modality-level SHAP attribution for the trimodal Text+Imaging+Audio XGBoost early-fusion model on the corrected fixed split.

Modality	Number of Features	Relative Mean |SHAP|
Text (SBERT)	384	**51.0%**
Audio (COVAREP)	148	**43.5%**
Imaging (OpenFace)	148	5.5%
**Total**	680	100%

S4.3. LIME Local Analysis

LIME provides a complementary local analysis of individual predictions. Table S8 reports the independently re-verified results for 30 explained participants from the corrected fixed split.

Table S8. Re-verified mean absolute LIME impact by modality for the bimodal XGBoost early-fusion model.

Class	Text	Landmarks	Gaze	Imaging/Text Ratio
Non-depressed	0.916	0.268	0.015	0.309
Depressed	0.918	0.297	0.034	0.360
**Overall**	**0.916**	**0.276**	**0.020**	**0.323**

The overall imaging/text ratio is therefore 0.323 under the re-verified LIME analysis. The considerably larger local imaging contribution relative to the global SHAP decomposition is not necessarily contradictory: SHAP summarizes global additive attribution in the fitted tree model, whereas LIME measures local sensitivity around individual predictions.

Taken together, the SHAP and LIME analyses indicate that imaging has limited global influence in the bimodal early-fusion XGBoost model but may contribute locally for selected participants. In the trimodal early-fusion model, SHAP assigns substantial global importance to the audio modality. Because neither analysis directly explains the final neural late-fusion ensemble, no final-model modality weights are inferred from these results.

S5. Computational Cost

Training time and inference latency were measured in CPU-only mode during the corrected pipeline. Because timings depend on hardware, software versions, batch composition, and fold size, they are reported as implementation-specific ranges rather than hardware-independent deployment benchmarks.

Table S9. Observed computational-cost ranges for the principal model families in the corrected participant-level pipeline. Training time denotes one train/validation fit; inference latency is reported per participant.

Model Family	Training Time/Fit	Inference/Participant	Execution Mode
Text MLP	0.4–2.4 s	< 10 ms	CPU
ANN+Attention	0.3–3.0 s	< 10 ms	CPU
CNN+LSTM+Attention	10–16 s	< 20 ms	CPU
XGBoost / LightGBM	< 1 s	< 5 ms	CPU

The complete five-fold trimodal evaluation, including modality-specific fitting, internal validation, threshold selection, and fusion, requires on the order of several minutes in CPU-only mode on the hardware used for this study. These timings indicate that participant-level inference is lightweight relative to feature extraction, but they should not be interpreted as formal real-time or clinical-deployment benchmarks.

S6. Additional E-DAIC Feature-Space Diagnostics

The following two tables are retained in the Supplementary Material because they support secondary interpretation of the E-DAIC-native feature space rather than the primary DAIC-WOZ model-comparison claim.

S6.1. Classical-Classi ier Sanity Check

Table S10. Supplementary out-of-fold AUC of E-DAIC-native imaging and audio descriptors using classical classifiers. Regenerated after correcting the PHQ-8 labeling inconsistency described in the main text. Values around 0.5 indicate limited standalone ranking ability.

Classifier	Imaging AUC	Audio AUC
Logistic Regression (balanced)	0.396	0.575
Random Forest (balanced)	0.530	0.534
Gradient Boosting	0.498	0.526

S6.2. Exploratory Adaptive-Fusion Comparison

Table S11. Supplementary exploratory AUC comparison of fusion rules on the pooled E-DAIC-native feature space. Regenerated after correcting the PHQ-8 labeling inconsistency affecting 20 of 275 pooled participants (15 development and 5 participant-disjoint evaluation participants; see main text).

Fusion Strategy	AUC
Unweighted averaging	0.650
Confidence-weighted fusion	**0.686**
Text-guided gating	0.662
Stacked fusion (logistic meta-learner)	0.671
